# Neuroimaging genetics approaches to identify new biomarkers for the early diagnosis of autism spectrum disorder

**DOI:** 10.1038/s41380-023-02060-9

**Published:** 2023-04-17

**Authors:** Sabah Nisar, Mohammad Haris

**Affiliations:** 1 Laboratory of Molecular and Metabolic Imaging, Sidra Medicine, Doha, Qatar; 2 Center for Advanced Metabolic Imaging in Precision Medicine, Department of Radiology, Perelman School of Medicine, University of Pennsylvania, Philadelphia, PA USA; 3 Laboratory Animal Research Center, Qatar University, Doha, Qatar; 4 Present Address: Department of Diagnostic Imaging, St Jude Children’s Research Hospital, Memphis, TN USA

**Keywords:** Diagnostic markers, Autism spectrum disorders

## Abstract

Autism-spectrum disorders (ASDs) are developmental disabilities that manifest in early childhood and are characterized by qualitative abnormalities in social behaviors, communication skills, and restrictive or repetitive behaviors. To explore the neurobiological mechanisms in ASD, extensive research has been done to identify potential diagnostic biomarkers through a neuroimaging genetics approach. Neuroimaging genetics helps to identify ASD-risk genes that contribute to structural and functional variations in brain circuitry and validate biological changes by elucidating the mechanisms and pathways that confer genetic risk. Integrating artificial intelligence models with neuroimaging data lays the groundwork for accurate diagnosis and facilitates the identification of early diagnostic biomarkers for ASD. This review discusses the significance of neuroimaging genetics approaches to gaining a better understanding of the perturbed neurochemical system and molecular pathways in ASD and how these approaches can detect structural, functional, and metabolic changes and lead to the discovery of novel biomarkers for the early diagnosis of ASD.

## Introduction

Autism spectrum disorder (ASD) is a neurodevelopmental disability that manifests in early childhood and is characterized by deficits in social skills, behaviors, and communication. According to the World Health Organization, approximately 1 in 160 children worldwide [1] and about 1 in 44 children in the United States have ASD [2], which can occur in all racial and ethnic groups, and is four times more prevalent in boys than in girls [3]. Individuals with ASD can have co-occurring conditions, such as attention deficit hyperactivity disorder (ADHD), bipolar disorder, depression, intellectual disability, language and developmental delays, speech disorder, and gastrointestinal symptoms [4]. Although the cause of ASD is ambiguous, genetic and non-genetic factors most likely contribute to its development [5].

ASD is associated with several genetic syndromes, a high incidence of chromosomal rearrangements, and the presence of common and rare variants [6]. Methodologic advances have revealed that common, heritable polygenic risk accounts for ~50% of ASD cases; major-affect mutations account for 15%; and rare de novo copy number variations (CNVs) and single-nucleotide variants (SNVs) that alter the structural genome account for ~5% [7]. No theory posits a clear unifying mechanism of ASD at the molecular or cellular level, because it remains unclear whether ASD is many disorders converging on a few molecular pathways or a few disorders with complex, diverse mechanisms [8].

The cellular and molecular bases of autism can be attributed to increased local connectivity in brain regions, neuronal migration deficits, excitatory/inhibitory imbalance, and synaptic dysregulation [9–12]. Many studies have highlighted the genetic heterogeneity underlying ASD and indicated that several ASD-associated gene or protein products interact with neuronal, synaptic, and other neurodevelopmental pathways [13, 14]. Neurologic disorders, such as ASD, cause microdamage to the brain, and detection of the resulting structural and functional changes requires the use of high-resolution, noninvasive imaging techniques, such as magnetic resonance imaging (MRI). Furthermore, neuroimaging studies have provided evidence of altered cortical and subcortical structures, impaired white matter (WM) connectivity, and atypical connectivity in the frontal and temporal brain regions involved in various cognitive functions [15].

Because genes directly affect brain development and function, genetic polymorphisms or aberrations might be strongly associated with the functioning of the compromised neural systems and behavioral outcomes [16]. Neuroimaging can be used to investigate the effect of genetic variations on brain structure, function, and connectivity; this approach is known as “neuroimaging genetics” [17]. Neuroimaging genetics can delineate the molecular mechanisms induced by genetic variants (common and rare) linked to neurodevelopmental disorders (NDDs). Neuroimaging genetics enables us to investigate gene-specific effects on different functional brain systems, which will contribute to future diagnosis of various NDDs, including ASD.

In this review, we will explore various neuroimaging techniques that can be used to assess the impact of genetic factors on brain structure, function, and metabolism. In addition, we will discuss the neuroimaging genetics approach can be used to identify novel biomarkers for the early diagnosis of ASD.

## Brain changes associated with genetic changes in ASD

### Structural and functional changes

Several gene polymorphisms have been linked to structural and functional changes in the brains of individuals with ASD. For example, Homeobox (*HOX*) genes that play an important role in defining cell identity and positioning during embryonic development are also associated with autism [18]. An earlier study has shown aberrant hindbrain development and craniofacial defects in *HOXA1/HOXB1*-mutant mice as a result of rhombomere misspecification within the hindbrain [19]. Other studies have shown the association of *HOXA1 A218G* polymorphism with increased head circumference in autistic individuals [20, 21]. On the other hand, *HOXB1* alleles have been found to affect stereotypic behaviors and influence head growth rates, but to a much lesser extent than *HOXA1 A218G* in autistic individuals [22]. Current research suggests that approximately 25% of individuals with constitutional *PTEN* mutations might meet the criteria for ASD [23]. Autistic individuals with germline *PTEN* mutations D252G (exon 7), H93R (exon 4), and F241S (exon 7) were found to have increased head circumferences than other autistic subjects [24]. In another study, the de novo missense *PTEN* mutation D326N (exon 8) was identified in an autistic patient with developmental delay, mental retardation, and extreme macrocephaly; the patient also showed prenatal and postnatal overgrowth [25].

A recent systematic review summarized brain structural MRI (sMRI) findings in monogenic disorders that are strongly associated with ASD [26]. The review included mutations in *PTEN*, *SHANK3*, *SYNGAP1*, *CHD8*, *ARID1B*, *ADNP*, *POGZ*, *MED13L*, *SLC6A1*, and *ANKDR11*, which are associated with different brain abnormalities, most prominently in the WM, GM, and ventricular regions [26]. Studies have also reported the association of contactin-associated protein 2 (*CNTNAP2*) polymorphisms with WM and GM abnormalities [27]. *CNTNAP2* is a master gene that causes speech-language delay and is central to the manifestation of autism [28]. A recent MRI study involving 118 individuals with ASD and 122 typically developing (TD) controls showed the association of a *CNTNAP2* variant (rs2538991) with WM volume of the right anterior cingulate gyrus in ASD individuals [29].

MET receptor tyrosine kinase and its ligand, hepatocyte growth factor help mediate neurodevelopmental events that are associated with brain structural pattern and circuitry and has a pleiotropic role in multiple organs’ ontogenesis [30]. Functional polymorphism in *MET* gene has been associated with increased risk for autism [31]. A study involving 75 individuals with ASD and 87 TD controls showed an ASD-risk variant in the *met receptor tyrosine kinase* gene to be associated with altered WM connectivity in individuals with ASD, relative to TD controls [32].

Mutations associated with the chromodomain helicase DNA-binding protein 8 (*CHD8*) gene are also implicated in autism. CHD8 is a transcriptional regulator that is involved in the remodeling of chromatin structure and is crucial for dendrite development and neuronal migration [33]. A case report study, using whole-exome sequencing (WES), identified a de novo mutation of the *CHD8* gene in a clinical ASD phenotype including intellectual disability (ID), macrocephaly, and craniofacial abnormalities observed in a boy with developmental delay [34].

In a study comparing mouse models of autism to wild-type (WT) controls, *NLGN3-* and *MECP2-*mutant mice showed increased cerebellar volumes, and *ITGB3-*mutant mice showed reduced cerebellar volume [35]. Functional analysis of cortical neurons in *MECP2*-mutant mice showed abnormal growth of dendrites and axons, suggesting that *MECP2* mutations impair neuronal development which might lead to ASD [36]. Mutations associated with the SH3 and multiple ankyrin repeat domains 3 (SHANK3), a synaptic scaffolding protein required for synaptic functioning, have been implicated in ASD, with knockout (KO) mice having reduced total brain volume, hippocampus, and thalami, and enlarged basal ganglia [37]. Loss of *SHANK3* has been associated with altered prefrontal functional connectivity in mice, suggesting that this deletion impairs social and communication behaviors and contributes to ASD pathogenesis [38].

Neurexins are presynaptic cell-adhesion proteins that are involved in synapse formation. Single-cell RNA sequencing analysis on induced pluripotent stem cells-derived neural stem cells revealed that, compared to neurons of a healthy patient, those of an autistic patient carrying the biallelic neurexin 1-alpha (*NRXN1-α*) deletion had impaired maturation of action potentials and decreased calcium signaling [39]. Additionally, diffusion MRI analyses of the brain tissues of *NRXN2-α–*KO mice showed altered microstructures in the social brain regions and impaired structural connectivity between the amygdala and orbitofrontal cortex regions, suggesting a role of *NRXN2* in altering social behaviors [40].

In another study, homozygous *CNTNAP2*^*–/–*^ mice exhibited reduced long-range and local functional connectivity in the prefrontal and midline brain regions, suggesting that homozygous loss-of-function mutations in *CNTNAP2* predispose individuals to NDDs, such as autism [41]. Another study using a forebrain organoid model generated from induced pluripotent stem cells of patients with syndromic ASD carrying the homozygous *CNTNAP2* c.3709DelG mutation showed that the mutation causes cortical overgrowth in the organoids that was rescued by repairing the pathogenic mutation via CRISPR–Cas9, thus confirming the causative effect of homozygous *CNTNAP2* mutation in ASD [42].

CD38, a transmembrane protein plays an important role in controlling social behaviors due to its role in regulating oxytocin secretion processes [43]. Two single-nucleotide polymorphisms (SNPs) in the *CD38* gene (rs3796863 and rs1800561) have been detected in individuals with ASD [44]. Plasma levels of oxytocin were lower in individuals with ASD carrying the R140W allele than in those lacking the allele [44]. Treating a proband carrying the R140W allele with intranasal oxytocin improved social, communication, and emotional behaviors [44].

Along with CD38, oxytocin receptor (*OXTR*) genes influence social behavior, and *OXTR* mutations are a risk factor for ASD [45]. Genotyping for SNPs and fMRI analysis of 38 adolescents with high-functioning autism (HFA) and 33 TD controls [46] showed *OXTR* SNPs association with brain activation within the right supramarginal gyrus and inferior parietal lobule during an emotion-recognition task in autistic individuals [46]. Another study involving 209 probands with ASD investigated the influence of two polymorphisms (rs1042778, rs53576) in *OXTR* on ASD-related clinical symptoms including panic and aggressive behaviors [47]. The presence of *OXTR* rs1042778 T allele was associated with panic and aggressive behaviors in individuals with ASD, suggesting the importance of *OXTR* in ASD diagnosis and clinical phenotypes [47]. The disparity in the prevalence of ASD among males and females gives rise to a sex bias, a concept that is poorly understood in the neurobiology of autism. In relation to this, an imaging-genetics study consisting of 50 females with HFA and 52 females as TD controls and 37 males with HFA and 34 males as TD controls between the ages of 8 and 17 assessed the impact of ASD-associated *OXTR* variants on reward network functional connectivity in both male and female subjects with ASD [48]. Females carrying more ASD-associated *OXTR* variants showed increased connectivity between reward-related brain regions (nucleus accumbens) and the prefrontal cortex region, compared to males with ASD [48].

Genetic variants of arginine vasopressin receptor 1 A (*AVPR1A*) gene have also been linked with autism [49]. In a study involving 121 healthy volunteers, a functional imaging task was performed to assess the association between *AVPR1A* genetic variants and amygdala activation [49]. Carriers of the *AVPR1A* ASD-risk alleles (RS1 and RS3) had differential activation of the amygdala [49]. In another study involving 1104 healthy subjects, MRI, genotyping, and learning and memory assessment were performed to assess the effect of *AVPR1A* RS3-RS1 haplotypes on verbal learning and memory: individuals carrying the short alleles of RS3-RS1 haplotypes displayed poor verbal memory performance than those carrying the long alleles [50]. A study consisting of 212 ASD probands and their biological parents conducted a family-based association test to assess the effect of polymorphisms in the *AVPR1A* promoter region on social behavior [51]. The study found two *AVPR1A* SNPs (rs7294536 and rs10877969) to be over-transmitted as a risk allele in Korean families with ASD; suggesting their involvement in dysregulating social behavior and contributing to the pathophysiology of ASD [51].

Mutations associated with the reelin (*RELN*) gene, which encodes a large glycoprotein RELN that guides neuronal migration and positioning during embryonic development [52], have been implicated in ASD pathology [53, 54]. A de novo RELN R2290C mutation identified in an ASD proband in a conserved arginine-amino acid-arginine domain were found to impair RELN protein secretion and these effects were recapitulated in a heterozygous *RELN* mouse mutant model [55]. Analysis of *RELN* R2290C heterozygous neurospheres revealed an upregulation in Protein Disulfide Isomerase A1, a chaperone protein responsible for the formation of disulfide bonds [55]. In contrast, a study comparing plasma RELN levels between 40 ASD and 19 healthy children found higher RELN levels in children with ASD than in healthy controls [56].

The gene T-brain 1 (*TBR1*) encodes the transcription factor TBR1, which mediates gene transcription and cortical neurogenesis and is crucial for normal neurodevelopment. Several preclinical and clinical studies have shown *TBR1* to be implicated in ASD [57–61]. *TBR1* haploinsufficiency results in defective axonal projections of amygdala neurons and impairs social interaction, memory, cognitive flexibility [58], and neuronal activation of the olfactory system in mice models [57]. Additionally, mice carrying the heterozygous *TBR1 K228E* mutation showed altered cortical development, increased levels of TBR1, inhibitory synaptic transmission, and ASD-like behavioral phenotypes [60]. De novo and missense *TBR1* mutations also disrupt TBR1 functions, such as subcellular localization and transcriptional repression, in individuals with sporadic ASD [61]. Moreover, TBR1 interacts with FOXP2, which is associated with speech and language disorders and the TBR1–FOXP2 interaction was shown to be abolished in patients with sporadic ASD [61].

Ankyrin 2 (*ANK2*), an important gene that encodes for ankyrin B (ankB) protein is involved in membrane stabilization and localization of ion channels and transporters. *ANK2* mutation in mice has been found to increase axon branching and ectopic connectivity and impairs social and communication behaviors [62]. *ANK2* is highly expressed during early neurodevelopmental stages and is an important regulator of neurogenesis [63]. Loss of *ANK2* impaired differentiation of neural stem cells to neurons and altered the expression of genes involved in neural development, suggesting *ANK2* haploinsufficiency as a risk factor for ASD [63].

Mice carrying the *NLGN3 R351C* mutation showed delayed synapse elimination in the cerebellum suggesting NLGN3 involvement in synapse refinement of the cerebellar circuitry that might be associated with ASD pathogenesis [64]. Additionally, *NLGN3 R451C*–mutant mice displayed more aggressive and repetitive behaviors than WT controls [65]. Another study observed that *NLGN3*-knockin mice exhibited reduced GM and WM volumes and reduced social and anxiety-related behaviors compared to WT controls [66].

These studies provide evidence that monogenic risk factors for autism share common involvement of the prefrontal cortex, cerebellum, amygdala, and hippocampus. In addition, most of the genetic mutations responsible for structural and functional brain changes converge on biological pathways that are involved in corticogenesis, synaptogenesis, chromatin modification, and transcriptional and translational processes, all of which contribute to social and behavioral impairments, the core deficits associated with ASD.

### Metabolic changes

Proper regulation of cellular metabolism is essential for maintaining cellular function, which is crucial for the central nervous system (CNS), specifically for the brain, where energy consumption and metabolic changes are dynamic [67], and metabolic changes in neurons are critical for neuroplasticity and cognitive functions [67]. The heterogeneous and multifaceted pathological nature of ASD clearly explains why the genes affecting brain metabolism have been under-investigated. Nevertheless, to refine the search for metabolic biomarkers in ASD, the effect of candidate genes involved in ASD must be explored in metabolic pathways specifically affecting brain energy metabolism and neuron-astrocyte interactions to provide insight into which metabolic pathways are disrupted in ASD and how to target those pathways via neuroimaging genetics.

Autism is a multifactorial disease, with many candidate genes in its etiology. However, only a few of those genes such as such as *DISC1*, *SHANK3*, *ITGB3*, *SLC6A4*, *RELN*, *RPL10*, and *AVPR1α* are found to be associated with brain metabolism [68]. Moreover, several metabolic pathways are found to be altered in the prefrontal cortex of ASD individuals [69]. These metabolic pathways include glutathione metabolism, galactose metabolism, purine and pyruvate metabolism, starch and sucrose metabolism, arginine and proline metabolism, cysteine and methionine metabolism, propanoate metabolism, nicotinate, and nicotinamide metabolism, and the tricarboxylic acid (TCA) cycle [69].

The RNA-binding fox 1 (*RBFOX1*) gene is associated with various neuropsychiatric disorders (e.g., ASD, ADHD, epilepsy, intellectual disability, and schizophrenia) [70–73]. A study has also highlighted the role of cytoplasmic RBFOX1 in regulating the expression of genes involved in synaptic transmission and autism [74]. No association of *RBFOX1* with brain metabolism in ASD has been found, but its role in Alzheimer’s disease suggests its exploration in ASD.

Mitochondrial dysfunction is one of the most common metabolic abnormalities in ASD [75], and lactate and pyruvate are important biomarkers for mitochondrial energy metabolism [76]. Many studies have shown that lactate dehydrogenase A (*LDHA*) and B (*LDHB*) are involved in ASD pathophysiology [77–81].

Glucose fuels neuronal oxidative metabolism by providing ATP and the precursors required for neurotransmitter synthesis; glucose is also transported across the blood–brain barrier and into neurons by facilitative glucose transporters [82]. Glucose transporter-1 (GLUT-1) is predominantly found in the endothelial cells of the blood–brain barrier; GLUT-3 is the main transporter expressed in neurons [83]. Increased mRNA levels of GLUT-1, GLUT-3, and three key enzymes in glucose metabolism (hexokinase 1, pyruvate kinase, and pyruvate dehydrogenase) have been observed in the contralateral brain area of a mouse model of traumatic brain injury (TBI) [84]. Additionally, increased expression of lactate transporter in astrocytes and reduced expression of neuronal MCT-2 has been observed in the ipsilateral cortex and hippocampus of the TBI mouse model, suggesting that sustained impairment of glucose metabolism after TBI is neuron-specific [84]. Neuronal *GLUT-3*-deficient heterozygous mice demonstrated ASD-like features, and this phenotype was associated with increased GLUT-1 and MCT-2 concentrations suggesting that the neuronal glucose deficiency was compensated for enhanced uptake of lactate by the brain [85]. Despite this metabolic compensation, neuronal function was altered in *GLUT-3*–heterozygous mice, as observed by increased electroencephalographic seizure activity, neurobehavioral abnormalities (i.e., abnormal spatial learning and working memory), and deficits in social behavior, all features observed in ASD [85, 86]

In the adult CNS, cholesterol is derived through de novo synthesis by astrocytes [87]. A major constituent of cholesterol catabolism is the neuronal enzyme cytochrome P450 family 46 subfamily A member 1 (*CYP46A1*), which protects neurons and helps convert cholesterol to 24-hydroxycholesterol (24 HC), enabling it to cross the blood-brain barrier [88]. An indirect association of CYP46A1 has been shown in autistic children with high plasma levels of 24 HC [89]. Moreover, 24 HC plasma levels have been inversely correlated with age in autistic individuals [89].

The disrupted in Schizophrenia 1 (*DISC1*) gene is involved in neurodevelopmental processes, such as neuronal proliferation, differentiation, and migration [90]. Prenatal disruption of *DISC1* in fetal neural progenitor cells of the dominant-negative *DISC1* (DN-DISC1) adult mice has been found to cause significant anxiety and depression-like behavioral changes [91], elevated levels of GABA, and increased cell density of parvalbumin^+^ interneurons in the cingulate cortex, motor cortex, and the retrosplenial granular cortex [91]. Alternatively, somatostatin^+^ and neuropeptide-Y^+^ interneurons were found to be decreased in other brain regions, suggesting that disrupting DISC1 function affects the localization of interneuron subtypes [91]. Moreover, DN-DISC1 was found to interact with Dlx2 and negatively regulate Dlx2-mediated Wnt-signaling pathway activation [91]. Knocking down *DISC1* and the expression of DN-DISC1 was found to decrease GLUT-4 mRNA and protein levels and reduce glucose uptake by primary astrocytes, which was associated with reduced oxidative phosphorylation, glycolysis, and lactate production in vitro and in vivo [92]. Moreover, treatment with lactate rescued the behavioral abnormalities in DN-DISC1 mice [92]. Thus, altered *DISC1* function in astrocytes contributes to metabolic abnormalities that might cause cognitive and behavioral deficits in various neuropsychiatric disorders [92]. In a recent study, deletion of *AUTS2* was found to impair social interactions, reduce uptake of brain glucose, and inhibit the pentose phosphate pathway in a conditional knockout mouse model with *AUTS2* deletion (*AUTS2*-cKO) [86].

Obesity is a common risk factor associated with NDDs, such as ADHD and ASD [93, 94]. The adolescent medial prefrontal cortex region is found to be vulnerable to high-fat diets (HFDs) via *RELN* deficiency [95]. *RELN* acts through ApoER2 and very-low-density lipoprotein receptor (VLDLR) and plays an important role in cholesterol and fatty acid metabolism [96]. Hypothalamic levels of RELN protein and APoER2 and VLDLR mRNA were found to be altered in mice fed with HFD [97]. Moreover, the recombinant central fragment of RELN affects membrane potential and action potential firing by altering pre- and postsynaptic inputs on the arcuate nucleus satiety-promoting proopiomelanocortin (ARH-POMC) neurons in a POMC-EGFP mouse model, thus suggesting the role of RELN in mediating energy homeostasis by acting on ARH-POMC neurons [97].

The role of SHANK3 in synaptic function and development and its recognition as an important candidate gene in ASD has been well established [98], but that in cerebral metabolism has not been well studied. Increased rates of cerebral synthesis have been reported in several brain regions of the *SHANK3-*KO mice, thus indicating high protein turnover [99]. Moreover, increased pERK in hippocampal tissues, and reduced pERK/ERK and pmTOR/mTOR ratios in synaptosomal-enriched frontal cortex lysates suggested a loss of protein-synthesis regulation via these pathways [99].

The heterogeneous and multifaceted pathological nature of ASDs clearly explains why the genes affecting brain metabolism are under-investigated. To refine the search for metabolic biomarkers in ASD, the effect of candidate genes involved in ASD must be explored in different metabolic pathways specifically affecting brain energy metabolism and neuron-astrocyte interactions. Identification of these metabolic alterations in the brain can provide an insight into the metabolic pathways disrupted in ASD and help target those pathways using the “neuroimaging genetics” approach.

## Role of imaging in detecting brain changes

Neuroimaging is vital to understanding the structure and functioning of the brain. In the field of ASD, most neuroimaging genetics studies are focused on structural and functional brain assessments. In this review article, we emphasize the imaging techniques used for monitoring changes in brain metabolism and neurotransmitter levels in individuals with ASD.

### Imaging for metabolic changes

Altered or impaired metabolic pathways are characteristic of many neurological disorders. Targeting these altered metabolic pathways non-invasively using PET and MRI provides a better understanding of the metabolic profile in neurological disorders, which can facilitate diagnosis and evaluations of therapeutic response. Cerebral glucose, fatty acid, lactate, and mitochondrial metabolism are altered in ASD pathophysiology [100–103]. To image glucose metabolism in the brain, ^18^F-FDG PET is most commonly used and determines the transport rate of glucose from the blood to the brain by a carrier-mediated diffusion mechanism [104]. Many studies have used ^18^F-FDG–PET to study cerebral glucose metabolism in ASD [105–108].

In addition to PET, magnetic resonance (MR) techniques have been applied to track brain metabolism. Magnetic resonance spectroscopy (MRS) is a widely known technique that has been used to identify molecular abnormalities associated with ASD. Using the magnetic properties of hydrogen, the noninvasive ^1^H-MRS technique generates a frequency spectrum that can identify different metabolites [109]. The ^13^C-MRS technique is widely used to measure neurotransmission and cell-specific neuroenergetics. The infusion of ^13^C-labeled substrates, such as glucose and acetate, enables the detection of rates at which ^13^C-labeled substrates are incorporated into cell-specific pools [110]. Due to the specificity of ^13^C-MRS, it is used to measure fluxes in the TCA cycle and the total neurotransmitter cycle. The measurement of TCA flux reflects the mitochondrial health of neurons and glia, and the ^13^C-MRS technique can measure the TCA flux in glia and glutamatergic and GABAergic neurons [110].

To maintain constant levels of high-energy phosphate compounds in the brain, the mitochondrial production and utilization of ATP are tightly regulated. The ^31^P-MRS technique is used to measure ATP synthesis; however, only two studies have reported its use to study metabolic dysregulation in the brains of individuals with ASD [111, 112]. In one study, brain high energy phosphate and membrane phospholipid metabolism were investigated in the dorsal prefrontal cortex of 11 autistic participants and 11 healthy controls using in vivo ^31^P-MRS [111]. The study observed decreased levels of phosphocreatine (PCr) and esterified ends (αATP + αADP + dinucleotides + diphosphosugars) and enhanced degradation of brain membranes in autistic subjects compared to controls, reflecting altered brain bioenergetics in autism [111]. Another study similarly evaluated muscle and brain energetics in 6 autistic cases and 6 healthy controls using in vivo ^31^P-MRS and observed decreased frontal PCr in cases compared to the controls [112].

Emerging evidence shows altered fatty acid metabolism and homeostasis in ASD [113]. Acetate is a marker of glial metabolism and a metabolic substrate of astrocytes during fatty acid synthesis [114, 115]. In recent decades, the PET tracer ^11^C-acetate has gained notoriety due to its application in imaging tumor lipid metabolism [116]. Its application in neurological disorders is still in its infancy, but ^11^C-acetate has been used to detect pathological changes in multiple sclerosis [117, 118] and mild cognitive impairment [119].

**Fig. 1:**
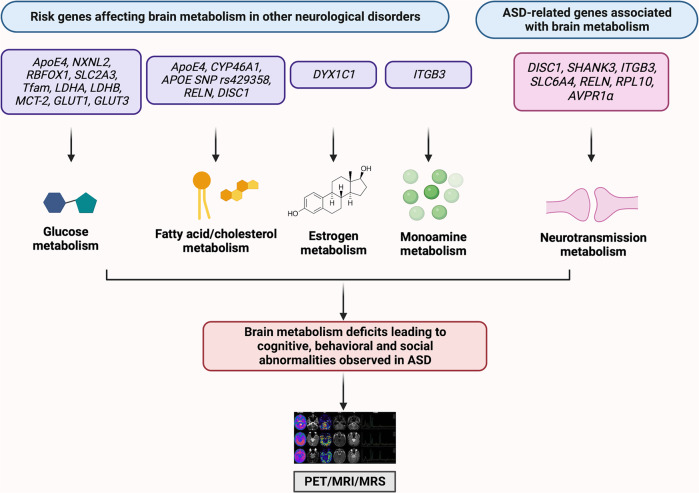
Genes associated with brain metabolism in autism-spectrum disorder (ASD) and other neurological disorders. Risk genes for various neurological disorders and ASD-related genes that affect brain metabolism can be detected by using neuroimaging techniques, such as positron emission tomography (PET), magnetic resonance imaging (MRI), and magnetic resonance spectroscopy (MRS).

Alterations in risk genes associated with ASD and other neurological disorders that affect glucose, fatty acid/cholesterol, estrogen, monoamine, and neurotransmission metabolism in the brain can be detected via PET, MRI, and MRS (Fig. 1). These techniques enable the identification of altered metabolic pathways and reveal potential metabolic biomarkers that possess translational potential to better diagnose neuropsychiatric disorders.

### Imaging for neurotransmitters

Neurotransmitters enable communication between neurons and non-neuronal cells via synaptic transmission and are essential to brain development and functioning. Dysfunction of the neurotransmitter system can affect neuronal migration, cell differentiation and proliferation, and synaptogenesis, thereby ultimately affecting brain development [120]. Dysregulated neurotransmitter systems are associated with the pathogenesis of various neuropsychiatric disorders, including ASD. The most common neurotransmitter systems associated with the pathogenesis of ASD are the serotonergic [121], dopaminergic [122], glutamatergic [123], and GABAergic [124] systems.

The neuromodulator dopamine acts as a reward center for the brain and regulates motivational and cognitive-behavioral control [125]. Mutations in dopamine transporters and receptors are associated with ASD pathophysiology [126–128]. The radiolabeled analog of L-DOPA, [^18^F]-L-dihydroxyphenylalanine (^18^F-FDOPA), is used to evaluate the central dopaminergic function of presynaptic neurons, including dopamine synthesis and transport via PET [129]. ^18^F-FDOPA–PET has been used to examine striatal dopamine synthesis capacity in ASD [130, 131]; [^11^C]WIN-35,428 PET to image dopamine transporters in high-functioning individuals with autism [132]. PET probes, such as [^11^C]NMS, [^11^C]SCH23390 PET, and [^11^C]raclopride PET, have been used to study dopamine receptor binding in individuals with ASD [133–135].

The inhibitory neurotransmitter serotonin modulates various developmental processes, such as neuronal migration, cell differentiation, cell division, and synaptogenesis [136]. Mutations in the serotonin transporter gene *SLC6A4* are associated with ASD pathology [137–141]. Tryptophan is an essential amino acid and precursor of serotonin that regulates emotional processes [142]. The dysregulation of tryptophan metabolites is associated with many neurological disorders [143–146], including ASD [147]. The PET probe AMT (α-[^11^C]methyl-L-tryptophan) is used to trace tryptophan metabolism via the serotonin or kynurenine pathways. AMT has been used to measure AMT uptake in autistic individuals [148]. Serotonin transporters regulate serotonergic neurotransmission by terminating the action of serotonin, removing it from synaptic clefts after its release [149]. Anomalies associated with serotonin transporters are well-reported in autism [150–153]. The PET probes used for detecting serotonin-transporter dysfunction in ASD are [^11^C]DASB [154, 155], [^11^C]MADAM [156, 157], and [^11^C]( + )McN5652 [132, 158]. Additionally, alterations in the serotonin receptors 1 A and 2 A have been associated with ASD [155, 159, 160]. One of the studies utilized [^11^C]DASB to compare serotonin transporter availability in 17 Asperger’s subjects and 17 healthy controls, finding no statistical difference in the groups’ regional binding potential of [^11^C]DASB [155]. Studies have also shown the reliability of [^11^C]MADAM in measuring serotonin binding in different regions in the brain [156, 157]. [^11^C]MADAM has been utilized as a radioligand for measuring serotonin transporter availability in the brain of 15 ASD subjects and 15 healthy controls [156], finding lower serotonin availability in the brain of ASD subjects compared to controls [156]. Moreover, using [^11^C]( + )McN5652, a potent serotonin uptake blocker, as a radiotracer to determine changes in the binding of serotonin transporter in autistic subjects revealed significantly less binding in autistic subjects than in controls [132].

GABA is an important inhibitory neurotransmitter in the CNS that regulates brain rhythm and neuronal activities during neurodevelopment. Mutations in GABA receptor genes have been associated with ASD [161–163]. Several studies have used [^11^C]RO15-4513–, [^18^F]flumazenil– and [^11^C]flumazenil–PET to measure the levels of GABA receptor subtypes and their densities in individuals with ASD [164–166]. Flumazenil is a competitive antagonist at the benzodiazepine binding site on the GABA_A_ receptor [167]. A study utilized [^18^F]flumazenil to measure GABA_A_ receptor densities in 28 autistic individuals and 29 healthy controls but reported no significant difference in the GABA_A_ receptor densities between autistic individuals and healthy controls [164]. Similarly, using [^11^C]flumazenil as a radiotracer to measure GABA_A_ receptor availability, a study reported no difference in GABA_A_ receptor availability between 15 ASD individuals and 15 healthy controls and in mouse models of ASD [165]. In another study, PET radioligand [^11^C]RO15-4513 was utilized to measure α1 and α5 subtypes of the GABA_A_ receptor levels in the brains of 3 HFA individuals and 3 healthy controls [166]. The study reported significantly lower binding of [^11^C]RO15-4513 driven by lower levels of GABA_A_ α5 subtype in autistic individuals compared to controls [166].

Glutamate is a major excitatory neurotransmitter that plays an important role in synaptic plasticity, which is essential for learning and memory, and the formation of neural networks during development [168]. Alterations in the glutamatergic system have been documented in various neuropsychiatric disorders, including ASD. We discussed the genetic mutations associated with glutamate and its receptors in ASD in a recent review [169]. The following PET probes are used to image the metabotropic glutamate receptors (mGLURs) in ASD studies: [^18^F]FPEB binds mGLUR5 [170], [^11^C]ITMM binds mGLUR1 [171], and [^11^C]MMPIP binds mGLUR7 [172]. However, only [^18^F]FPEB has been applied in most preclinical and clinical ASD imaging studies [173–175]. [^18^F]FPEB is a radiopharmaceutical that is used for the quantification of mGLUR5. In an in vivo study, [^18^F]FPEB was utilized to measure changes in metabotropic glutamate receptor 5 (mGLUR5) expression in a *SHANK3*-knockout mouse model (Shank3B^−/−^) [173]. The study reported increased binding potential of [^18^F]FPEB in the hippocampus, thalamus, and amygdala of Shank3B^−/−^ mice compared to control mice which was consistent with the increased expression of mGLUR5 in these brain regions [173]. In another study [^18^F]FPEB was employed for the determination of mGLUR5 binding in 6 autistic individuals and 3 healthy controls [174]. The study reported increased binding potential of [^18^F]FPEB in the postcentral gyrus and cerebellum of autistic individuals than in controls [174]. Similarly, using [^18^F]FPEB, a study measured the density and distribution of mGLUR5 in individuals with idiopathic ASD, fragile X syndrome (FXS) and TD controls [175]. The study found increased expression of mGLUR5 in the cortical regions of individuals with idiopathic ASD and reduced mGLUR5 expression in all the brain regions of individuals with FXS than in controls [175]. ASD-associated studies have employed PET probes targeting serotonin receptor 1 A [^18^F]MPPF [176] and those targeting serotonin receptor 2 A, which include [^18^F]Setoperone [177, 178] and [^11^C]MDL100907 [155]. A study employed [^18^F]Setoperone, a high affinity ligand, for the PET imaging of serotonin-2 (5-HT2) receptors in 6 HFA adults and 10 healthy controls and reported less thalamic binding potential of [^18^F]Setoperone in autistic individuals compared to controls [177]. Another study employed [^18^F]Setoperone as a radiotracer for imaging cortical 5-HT2 density and reported lower cortical 5-HT2 density in parents of ASD children (*N* = 19) compared to controls (*N* = 17) [178]. [^18^F]MPPF is a PET tracer that is a specific serotonin 5-HT(1 A) receptor antagonist and has been used to examine the association among 5-HT(1 A) receptor, GM volume, and social personality in 18 ASD individuals and 24 TD controls [176]. The study reported a deregulation of 5-HT(1 A) receptor density in the striatum of individuals with ASD, suggesting it as a contributing factor to their social disturbances [176]. Another study used radiotracer [^11^C]MDL100907 to characterize of 5-HT(2 A) receptor of 17 individuals with Asperger’s disorder and 17 healthy controls but reported no difference in the regional binding potential of [^11^C]MDL100907 of the two groups [155]. Additionally, a few studies have used the ^1^H-MRS technique to measure the glutamate/GABA ratio in mouse models of ASD [179, 180]. One study reported decreased glutamate/GABA ratio in the prefrontal cortex of *CNTNAP2*^–^/^–^ mice, which correlated with a social behavior deficit in a mouse model of ASD [179]. Another study reported increased glutamate content in the hippocampus of valproic acid– and thalidomide–induced rat models of autism, suggesting glutamatergic dysfunction in the pathogenesis of autism [180].

Acetylcholine is a neurotransmitter that modulates different cognitive functions and behaviors, such as attention, learning, memory, and motivation [181]. In the brain, acetylcholine can influence synaptic transmission and alter neuronal excitability [182]. Few studies have reported genetic mutations associated with neuronal nicotinic acetylcholine receptor in ASD [183, 184], and only one PET study has applied [^11^C]MP4A-PET to measure acetylcholinesterase activity in 20 individuals with autism and 20 healthy controls [185]. The study reported a significant reduction in acetylcholinesterase activity in the bilateral fusiform gyri of individuals with ASD than in controls [185].

**Fig. 2:**
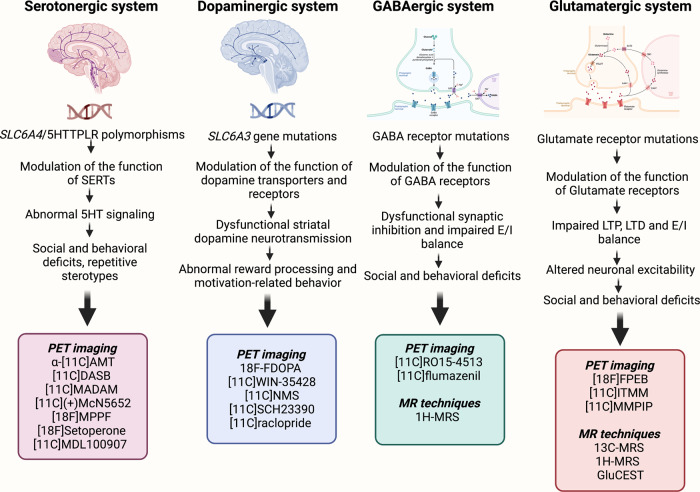
Neuroimaging techniques to detect serotonergic, dopaminergic, GABAergic, and glutamatergic abnormalities in autism-spectrum disorder (ASD). ASD-associated mutations in serotonin and dopamine transporters and in GABA and glutamate receptors can be detected by using various positron emission tomography (PET) probes and magnetic resonance (MR) techniques.

Many studies have used the ^1^H-MRS technique to quantify the levels of excitatory and inhibitory neurotransmitters in ASD [109] and to study altered neurotransmitter metabolism in highly functioning autistic individuals [186]. ^1^H-MRS has been used to measure the occipital concentrations of neurotransmitters GABA and glutamate plus macromolecules relative to creatine in individuals with HFA (*n* = 15 and *n* = 13 respectively) and healthy controls (*n* = 17) [186]. The findings reported a significantly higher Glu/Cr and lower GABA + /Glu concentrations in individuals with HFA compared to controls [186]. The recently developed technique glutamate chemical exchange saturation transfer (GluCEST) is used to image parenchymal glutamate in the brain and provides higher sensitivity and spatial resolution than ^1^H-MRS [187–189]. GluCEST imaging has been used in various neurological disorders [188, 190–193]. So far, no GluCEST imaging studies have been reported in individuals with autism. This imaging technique can serve as a diagnostic tool to identify dysfunctional glutamatergic systems and develop drugs or agents to rescue glutamate-associated behavioral deficits in ASD [169]. The information gleaned from the above studies show that alterations in the serotonin and dopamine transporter genes, and glutamate and GABA receptor genes that are responsible for causing social and behavioral phenotypes associated with ASD can be detected by employing different PET probes and MR techniques (Fig. 2).

## Integration of neuroimaging with genetics for the discovery of novel biomarkers

The neuroimaging genetics field emerged in the early 2000s and has advanced with the development of state-of-the-art imaging and genomic technologies. Initial studies used a candidate-gene approach to investigate the behavioral phenotypes associated with genetic polymorphisms [194, 195]. Progressively, other approaches emerged, including candidate gene–gene and candidate gene–environment interaction studies and the GWAS approach emerged, which required increased statistical power to detect variants and ultimately led to the development of multivariate approaches.

Advances in genomic technologies (e.g., whole-exome and whole-genome sequencing) allow a deeper investigation into the genome, and integrating neuroimaging with the latest genomic approaches can refine associations between the brain and genes and localize genetic effects to specific tissue layers rather than global structural volumes [196]. Moreover, neuroimaging genomics appears to be more advantageous in population-based cohorts rather than in patients because it enables identification of causal molecular processes involved in structural and functional brain changes without disease-related secondary effects.

The aim of integrating neuroimaging with genomic approaches is to delineate the association between genetic markers (e.g., SNPs, CNVs, epigenetic information, and other quantitative traits) extracted from neuroimaging data. The neuroimaging genetics approach has allowed researchers to not only identify the effects of risk-gene variants on brain morphology and function in ASD but also characterize the neural systems that directly affect those variants in ASD pathophysiology. In recent years, many neuroimaging genetics studies have examined genetic variants associated with ASD [27, 49, 197–200]. However, they have been limited by small sample sizes, a lack of reproducibility across cohorts, and a lack of GWAS data, which has hindered the identification of novel ASD-risk genes and cognitive phenotypes associated with ASD-risk variants. Thus, to overcome these limitations and expand the repository of genetic variants or pathways involved in ASD, we can apply a multimodal imaging approach in combination with the genotypic–phenotypic correlations to elucidate the underlying altered neural mechanisms in the pathophysiology of ASD. In addition, to characterize the complex genetic factors contributing to ASD, large sample sizes and advanced methods must be developed to examine the gene–gene and gene–environment interactions. Moreover, in the case of monogenic forms of ASD, a reverse-phenotyping approach can be applied to predict causative diagnosis for ASD [201]. Finally, for the translational success of neuroimaging genetics in ASD, more longitudinal imaging genetics studies are required to further validate the effect of specific genetic variants on behavioral phenotypes and to identify novel therapeutic targets or biomarkers.

## Machine learning to detect ASD biomarkers

ASD research is still in its infancy due to a lack of diagnostic tools. Clinicians rely on neuroimaging tools for the diagnosis of ASD, which can be achieved through neuroimaging-genetics studies and omics analysis combined with machine-learning (ML) tools. ML is a subfield of artificial intelligence (AI) that enables systems to “learn” and build an analytical model to predict outcomes and improve from experience, with minimal human intervention. ML uses different supervised learning methods (e.g., support vector machines, neural networks, gradient-boosting machine, random forest) to classify ASDs [202, 203]. These methods enable the machine to learn ASD-associated features and construct a relevant model that can be used for diagnostic purposes. Another type of ML is deep learning (DL), which is based on artificial neural networks inspired by information processing in the human brain. Countless studies have used deep neural networks (DNNs) and applied them to sMRI and fMRI data to diagnose ASD. To extract lower-dimensional features of ASD from fMRI, autoencoders (e.g., shallow, sparse, deep, and denoising) have been widely used [204–210]. The convolutional neural network (CNN) is another type of DL that evaluates visual information and can develop an internal representation of a 2D image. The CNN can be used to diagnose ASD, as it can extract features based on the convolutional layers [211]. Additionally, the CNN model includes normalization layers, fully connected layers, and pooling layers, which can interpret the diagnostic brain biomarkers in ASD by using fMRI [212]. DL is key to identifying complex patterns in high-dimensional fMRI data; thus, accurate designs of integrated methods or a multimodal architecture that combines features from high-dimensional sMRI and fMRI data can provide high accuracy and an efficient ASD diagnosis. Few studies have used CNN with resting state–fMRI data for the automated detection of ASD [212, 213]. One study used CNN with task-based fMRI for the fused global diagnosis of ASD [214], and another developed a DL-classification method based on 3D-CNN, with sMRI and fMRI imaging data integrated for the automated diagnosis of ADHD [215]. Moreover, the efficacy of DL methods can be enhanced by implementing predictive modeling and collaborative filtering techniques. Predictive modeling is a statistical technique that uses ML and data mining to forecast or predict future outcomes and can help promote the diagnosis of ASD [216, 217]. Collaborative filtering is another technique that can allow the creation of personalized recommendations based on the interactions, data, and preferences of other users in the system [218]. This technique can be used to build multi-model personalized recommendation ML algorithms that can facilitate the diagnosis of ASD [219].

One reason for the diagnostic delay of ASD is the increasing number of ASD evaluations requested, which increases the waiting time for families to meet with a specialist. Developing innovative AI-based technologies will help overcome these issues and augment various diagnostic aspects in ASD health care. A recent double-blinded cohort study tested the accuracy of an AI-based medical device that utilizes a gradient-boosted decision-tree algorithm to diagnose ASD [220]. For nearly one-third of the primary care sample, the AI-based device provided an efficient, highly accurate diagnostic evaluation [220]. Moreover, the CNN and DNN can be extended by using facial features as a physical biomarker for distinguishing autistic individuals from healthy controls. A recent study utilized five pre-trained CNN models and one DNN model to identify autistic individuals among TD individuals [221].

Unsupervised ML methods also have the potential to provide better diagnostic biomarkers for ASD. In the unsupervised ML technique, models are not supervised by training data sets; it uses ML algorithms to cluster and analyze untagged data sets. The unsupervised ML in ASD includes hierarchical clustering, *k*-means clustering, model-based clustering, and self-organizing maps [222]. Few studies have used unsupervised ML methods to predict short-term outcomes and explore cluster-based treatment efficacy in ASD [223, 224].

**Fig. 3:**
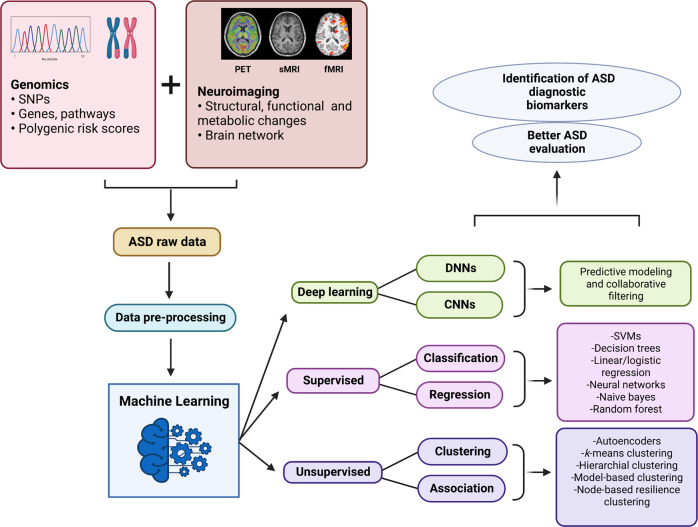
Machine-learning (ML) approaches for early diagnosis and better evaluation of autism-spectrum disorder (ASD). ML methods or techniques can be applied to ASD raw data obtained from genomics and neuroimaging approaches. ML uses supervised and unsupervised learning methods to classify and distinguish clusters in ASD. Deep-learning techniques, such as deep neural network (DNN) and convolutional neural network (CNN), can be applied to diagnose ASD.

Different studies have applied genomics data such as SNPs, spatiotemporal gene expression patterns, and common variants to predict ASD by using a machine-learning based approach [225–227]. Other studies have utilized data from neuroimaging techniques such as fMRI [228–232], sMRI [233] and PET [234] from ASD patients by implementing different ML tools to identify objective biomarkers, which is an important clinical goal in the management of ASD. The success of ML predominantly depends on the quality of data, features in the data, the choice of objective/loss function, and the selection of an appropriate model architecture that best fits the research question. Although, studies aiming for the discovery of novel diagnostic biomarkers for ASD have been advancing throughout the recent years, the application of ML tools using genomics and neuroimaging data in ASD is still in its infancy. Also, certain limitations are associated with the use of DL models in genomics that include model interpretation, curse of dimensionality, classification problems, data heterogeneity, and model tuning [235]. While in the neuroimaging data, sMRI and fMRI features are considered individually as predictors to ML models that affects the accuracy of the model. One approach that could improve the predictability and interpretability of the ML models is to use ASD raw data obtained from both genomics and neuroimaging studies and apply different ML algorithms (supervised learning, unsupervised learning, deep learning) that enable the identification of potential diagnostic biomarkers and provide a better evaluation for ASD (Fig. 3).

## Future perspectives

AI technology is relatively new in the ASD field, and more advances are needed to develop efficient predictive models for a better diagnostic experience. To diagnose autism, AI uses behavioral information from patients and performs data analysis to compare with other data sets. It then provides a tentative diagnosis enabling prediction of suitable therapeutic options for the affected individual. Considering the heterogeneity of ASDs, the functional and anatomical changes may occur simultaneously, which might prove difficult for accurate diagnosis. Moreover, the current ML and DL methods for ASD classification using 3D- and 4D-spatio-temporal data are still new and must be reformulated to increase the number of diagnostic parameters and overall running time. The fusion or combination of structural, functional, and metabolic imaging data with 3D- or 4D-CNN architecture can lead to the development of innovative classification tools that can improve the accuracy in the early diagnosis of ASD.
