# Correction to: Neuroimaging genetics approaches to identify new biomarkers for the early diagnosis of autism spectrum disorder

**DOI:** 10.1038/s41380-023-02115-x

**Published:** 2023-05-23

**Authors:** Sabah Nisar, Mohammad Haris

**Affiliations:** 1 Laboratory of Molecular and Metabolic Imaging, Sidra Medicine, Doha, Qatar; 2 Center for Advanced Metabolic Imaging in Precision Medicine, Department of Radiology, Perelman School of Medicine, University of Pennsylvania, Philadelphia, PA USA; 3 Laboratory Animal Research Center, Qatar University, Doha, Qatar; 4 Present Address: Department of Diagnostic Imaging, St Jude Children’s Research Hospital, Memphis, TN USA

**Keywords:** Diagnostic markers, Autism spectrum disorders

Correction to: *Molecular Psychiatry* 10.1038/s41380-023-02060-9, published online 17 April 2023

Previously, in the Figure 2, the boxes listing the PET probes for GABAergic and Glutamatergic system were switched mistakenly. Now we have corrected the mistake.
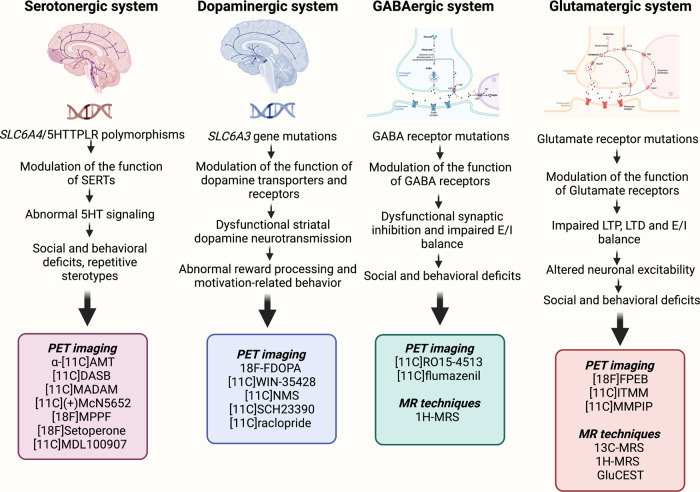


The original article has been corrected.

